# Synergistic Chondroprotective Effect of α-Tocopherol, Ascorbic Acid, and Selenium as well as Glucosamine and Chondroitin on Oxidant Induced Cell Death and Inhibition of Matrix Metalloproteinase-3—Studies in Cultured Chondrocytes

**DOI:** 10.3390/molecules15010027

**Published:** 2009-12-24

**Authors:** Anne-Christin Graeser, Katrin Giller, Heike Wiegand, Luca Barella, Christine Boesch‑Saadatmandi, Gerald Rimbach

**Affiliations:** 1 Institute of Human Nutrition and Food Science, Christian Albrechts University of Kiel, Hermann-Rodewald-Strasse 6, Kiel 24098, Germany; E-Mails: graeser@foodsci.uni-kiel.de (A.-C.G.); giller@foodsci.uni-kiel.de (K.G.); wiegand@foodsci.uni-kiel.de (H.W.); ch.boesch@foodsci.uni-kiel.de (C.B.-S.); 2 Bayer Consumer Care AG, Basel, Switzerland; E-Mail: luca.barella.lb@bayer.ch (L.B.)

**Keywords:** tocopherol, ascorbic acid, selenium, chondroitin, glucosamine, chondrocytes, oxidative stress, inflammation, metalloproteinases

## Abstract

Overproduction of reactive oxygen species and impaired antioxidant defence accompanied by chronic inflammatory processes may impair joint health. Pro‑inflammatory cytokines such as interleukin-1β (IL-1β) and tumor necrosis factor alpha (TNF-α) stimulate the expression of metalloproteinases which degrade the extracellular matrix. Little is known regarding the potential synergistic effects of natural compounds such as α‑tocopherol (α-toc), ascorbic acid (AA) and selenium (Se) on oxidant induced cell death. Furthermore studies regarding the metalloproteinase-3 inhibitory activity of glucosamine sulfate (GS) and chondroitin sulfate (CS) are scarce. Therefore we have studied the effect of α-toc (0.1–2.5 µmol/L), AA (10–50 µmol/L) and Se (1–50 nmol/L) on *t*-butyl hydroperoxide (*t*‑BHP, 100–500 µmol/L)-induced cell death in SW1353 chondrocytes. Furthermore we have determined the effect of GS and CS alone (100–500 µmol/L each) and in combination on MMP3 mRNA levels and MMP3 secretion in IL-1β stimulated chondrocytes. A combination of α-toc, AA, and Se was more potent in counteracting *t*‑BHP‑induced cytotoxicity as compared to the single compounds. Similarly a combination of CS and GS was more effective in inhibiting MMP3 gene expression and secretion than the single components. The inhibition of MMP3 secretion due to GS plus CS was accompanied by a decrease in TNF-α production. Combining natural compounds such as α-toc, AA, and Se as well as GS and CS seems to be a promising strategy to combat oxidative stress and cytokine induced matrix degradation in chondrocytes.

## 1. Introduction

Degenerative joint disorders including osteoarthritis (OA) and rheumatoid arthritis (RA) are characterized by an imbalance in the oxidant/antioxidant homeostasis resulting in oxidative stress, chronic inflammation, metalloproteinase activation and matrix destruction [1]. There is some evidence in the literature [2,3,4] for a beneficial role of antioxidant micronutrients on clinical symptoms of OA and RA. 

It has been shown that arthritis patients may exhibit decreased plasma α-toc levels and lower activity of the seleno-protein glutathione peroxidase (GSHPx) as compared to healthy control subjects [5]. Furthermore, a positive association between vitamin E consumption and joint health in humans has been recently reported [6]. However, litte is known about potential synergistic interactions of antioxidants including α-toc, AA and Se on arthritis prevention. Although the potential anti‑inflammatory and metalloproteinase-3 inhibitory activity of glucosamine sulfate (GS) and chondroitin sulfate (CS) has been studied using the single compounds *per se* [7,8,9], their combination has not been systematically investigated in cultured cells. Thus, it is largely unclear if and in what extent a combination of GS and CS may affect MMP3 in chondrocytes. The aims of this study were twofold: First we have systematically determined the potential synergistic effect of α‑toc, AA and Se on oxidant induced cell death in SW1353 chondrocytes. Secondly, we have investigated whether there is a synergistic inhibitory activity of GS and CS on MMP3 levels in chondrocytes stimulated with the pro‑inflammatory cytokine interleukin-1β (IL-1β).

## 2. Results and Discussion

### 2.1. Potential cytotoxic effects of the test substances and IL-1β

**Figure 1:**
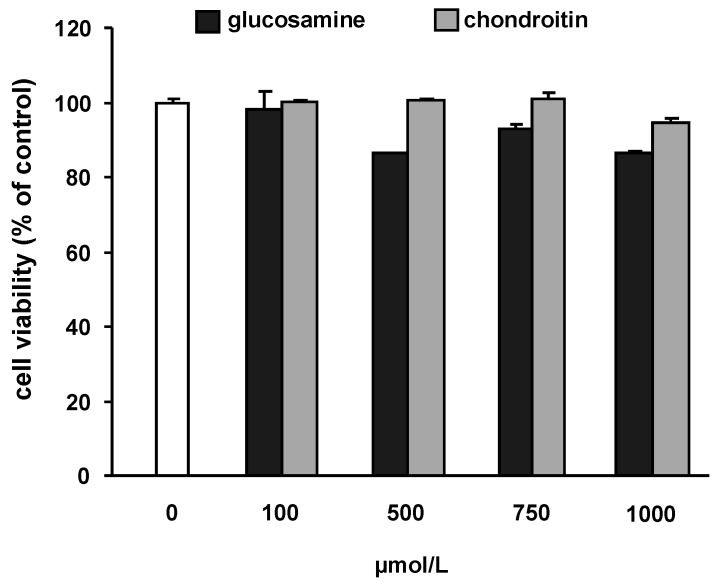
Effect of glucosamine and chondroitin on cell viability in SW1353 cells. Cells were treated with increasing concentrations of test substances for 24 h. The test compounds did not impair cell viability at any of the concentrations tested. Cytotoxicity was determined by the neutral red assay and cell viability is expressed as percentage of control (untreated cells).

To assure that glucosamine and chondroitin do not affect cell viability, cells were incubated with increasing concentrations of the test substances. As shown in Figure 1, cell viability of SW1353 cells was not significantly impaired up to a concentration of 1,000 µmol/L GS and CS, respectively. For all further experiments concentrations of 100 and 500 µmol/L of GS or CS were chosen. IL-1β was not cytotoxic up to a concentration of 100 ng/mL (data not shown). 

### 2.2. Effects of α‑tocopherol, ascorbic acid and selenium on tert‑butyl hydroperoxide induced cell death

**Figure 2:**
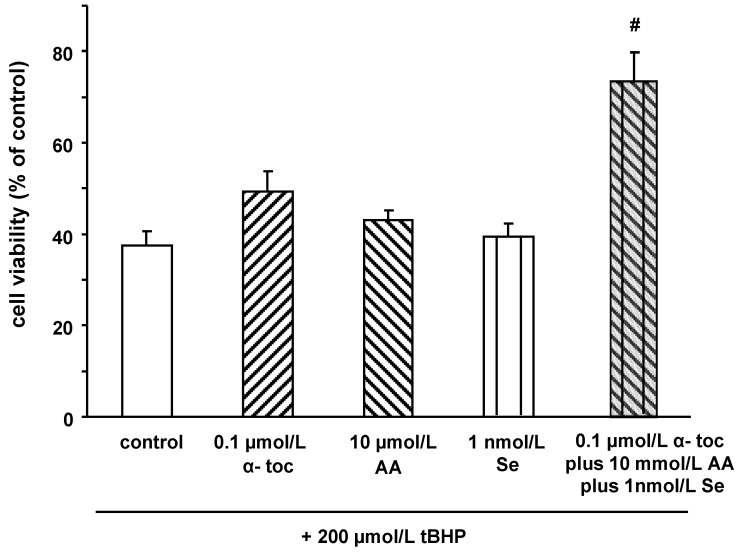
Effect of α-tocopherol, ascorbic acid, selenium, and their combination on cell viability in SW1353 cells after challenge with tBHP. Cells were treated with α-tocopherol (0.1 µmol/L), ascorbic acid (10 mmol/L), selenium (1 nmol/L), and a combination of all three test compounds (0.1 µmol/L α‑tocopherol, 10 µmol/L ascorbic acid, and 1 nmol/L selenium) for 24 h. After 3 h of challenge with 200 µmol/L *t*-BHP the neutral red assay was performed. Data are means + SEM of two independent experiments performed in duplicate. # *p *< 0.01; compared to control.

Treatment of SW1353 cells with increasing concentrations of *t*-BHP dose-dependently decreased cell viability, as summarized in Table 1. Pre-treatment of SW1353 cells with α‑toc, AA, and Se partly counteracted *t*-BHP induced cytotoxicity. Importantly, a combination of α‑toc, AA, and Se was more potent in counteracting *t*‑BHP induced cytotoxicity as compared to the single treatment with either of these micronutrients. Even at concentrations where the single component did not improve cell viability the combination of 0.1 µmol/L α‑toc, 10 µmol/L AA and 1 nmol/L Se was effective in partly preventing *t*-BHP-induced cell death (Figure 2). 

While 500 µmol/L *t*-BHP resulted in 70% cell death of SW1353 cells, the combination of 2.5 µmol/L α‑toc, 50 µmol/L AA and 50 nmol/L Se completely prevented t-BHP induced cytotoxicity (Table 1).

The generation of reactive oxygen species is an important factor in the development of human osteoarthritis. In fact, reactive oxygen species may damage lipids, proteins, and matrix components of chondrocytes [10]. Our data clearly indicate that a combination of α-tocopherol, ascorbic acid and selenium is more efficient in counteracting oxidant induced cell death than the single compounds *per se*. The radical scavenging activity of α‑toc is exerted through its phenolic hydroxyl group which readily donates a hydrogen to peroxyl radicals, resulting in the formation of stable lipid species [11]. Alpha-tocopherol becomes a relatively unreactive free radical as the unpaired electron becomes delocalized into the aromatic ring. The synergistic effect between AA and α‑toc, as observed in our study in chondrocytes, may be mediated by the matter of fact that the vitamin E radical is efficiently reduced from its free radical form due to AA that can directly regenerate the tocopheroxyl radical back to tocopherol [12]. 

Thus, in oxidant challenged chondrocytes, α‑toc and AA act synergistically to keep the steady state concentration of α‑toc high. Thereby a loss of consumption of vitamin E is significantly prevented.

**Table 1:** Effect of α‑tocopherol, ascorbic acid and selenium on cell viability after challenge with increasing concentrations of *t*-BHP. Data are means ± SEM and expressed in percentage of untreated control cells.

	*t*-BHP	100 µmol/L		200 µmol/L		500 µmol/L				
		**M**	***SEM***	**M**	***SEM***	**M**	***SEM***			
	***Control***	49.7	*4.0*	37.6	*3.2*	31.4	*1.2*			
	***α‑Tocopherol (α‑toc)*** ***µmol/L***									
	**0.1**	65.4	*5.4*	49.3	*4.6*	36.4^a^	*1.6*			
	**0.5**	90.7^c^	*3.8*	79.1^c^	*7.9*	64.8^c^	*3.3*			
	**2.5**	92.8^c^	*3.2*	89.7^c^	*1.4*	83.9^c^	*7.5*			
	***Ascorbic acid (AA) *** ***µmol/L***									
	**10**	60.8	*3.6*	43.1	*2.2*	32.4	*1.0*			
	**25**	78.2^c^	*2.6*	59.6^b^	*4.1*	36.1	*0.7*			
	**50**	73.0^b^	*4.5*	65.9^c^	*4.6*	37.5^a^	*1.8*			
	***Selenium (Se) *** ***nmol/L***									
	**1**	53.7	*4.3*	39.4	*3.1*	29.0	*1.2*			
	**10**	77.9^b^	*5.5*	55.4^b^	*4.6*	33.2	*1.2*			
	**50**	91.6^c^	*4.1*	83.0^c^	*1.7*	47.8^b^	*4.2*			
	***Combinations***									
	**I **(0.1 µmol/L α‑toc, 10 µmol/L AA, 1 nmol/L Se)	87.0^c^	*3.6*	74.2^b^	*6.1*	42.6^a^	*3.1*			
	**II **(0.1 µmol/L α‑toc, 25 µmol/L AA, 10 nmol/L Se)	98.1^c^	*3.2*	89.6^c^	*1.6*	69.0^c^	*4.6*			
	**III **(0.1 µmol/L α‑toc, 25 µmol/L AA, 25 nmol/L Se)	100.6^c^	*1.2*	95.2^c^	*2.4*	82.7^c^	*1.0*			
	**IV **(0.5 µmol/L α‑toc, 25 µmol/L AA, 10 nmol/L Se)	104.8^c^	*1.6*	97.8^c^	*2.8*	93.6^c^	*3.9*			
	**V **(0.1 µmol/L α‑toc, 50 µmol/L AA, 50 nmol/L Se)	99.3^c^	*2.7*	96.5^c^	*2.8*	94.4^c^	*2.3*			
	**VI **(0.5 µmol/L α‑toc, 50 µmol/L AA, 50 nmol/L Se)	101.7^c^	*4.1*	101.5^c^	*1.6*	94.8^c^	*2.0*			
	**VII **(2.5 µmol/L α‑toc, 50 µmol/L AA, 50 nmol/L Se)	103.0^c^	*1.2*	98.9^c^	*2.9*	98.0^c^	*4.5*			

^a^ *p *< 0.05; ^b^*p * < 0.01;^c^*p *< 0.001; different superscripts within a column indicate significant differences.

The protective effect of selenium on *t*-BHP-induced cell death in our chondrocyte model may be mediated due to the induction of the seleno-protein phospholipid‑hydroperoxide glutathione peroxidase (PHGPx). PHGPx is an antioxidant seleno-protein known to directly reduce phospholipid hydroperoxides in membranes, thereby interacting synergistically with vitamin E [13].

Taken together current data in cultured chondrocytes indicate that α‑toc, AA, and Se do not work in isolation from each other and are rather part of an interacting set of redox‑antioxidants [14]. As far as the concentration of the antioxidant nutrients are concerned, it needs to be taken into account that the concentrations of α‑toc (0.1–2.5 µmol/L), AA (10–50 µmol/L), and Se (1–50 nmol/L), as used in this study, are by in large physiologically achievable [15,16,17,18,19,20]. However the plasma concentrations of glucosamine [21] and chondroitin [22] may be lower as compared to the concentrations as used in our cell culture model.

### 2.3. mRNA levels and secretion of matrix metalloproteinase-3

**Figure 3:**
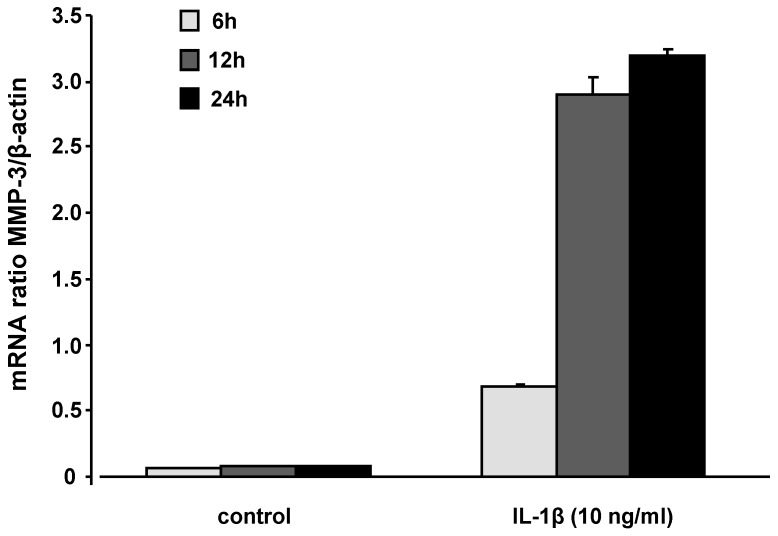
MMP3 mRNA levels in SW1353 cells following 6, 12, and 24 h of incubation with IL-1β (10 ng/mL). The highest induction of MMP3 was observed after 24 h of incubation. Results are calculated in relation to β-actin and expressed as means + SEM of three independent experiments performed in duplicate.

Reactive oxygen species may serve as intracellular signalling molecules that amplify the synovial inflammatory response [10]. The pro‑inflammatory cytokine IL-1β, as used in our SW1353 cell culture model, initiates multiple intracellular events that ultimately result in the activation of proteinases including matrix metalloproteinase-3 [23]. In this context, it is suggested that SW1353 cells may be a valuable cellular model to study the induction of protease expression by inflammatory cytokines, a phenomenon which is also evident in primary chondrocytes [24]. SW1353 cells were treated with IL-1β for 6, 12, and 24 h and MMP3 mRNA levels were determined by RT-PCR. MMP3 mRNA levels reached its maximum after 24 h of incubation (Figure 3), thus this time-point was selected for further MMP3 gene expression studies in the presence of the test compounds. 

MMP3 is a membrane bound zinc anti-peptidase which degrades extra‑cellular matrix [25]. Matrix metalloproteinase inhibitors have been proposed as potential therapeutic candidates in arthritis. 

**Figure 4:**
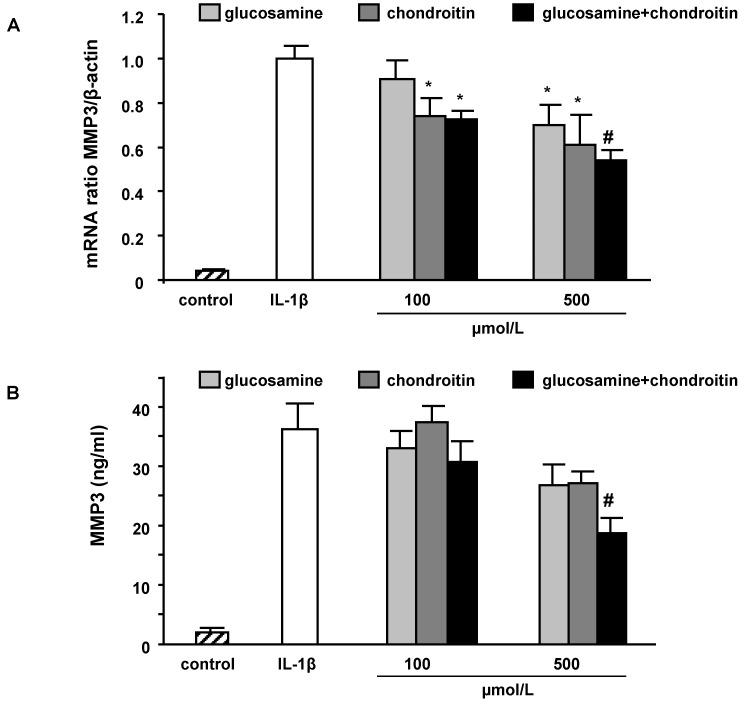
Effect of glucosamine and chondroitin on MMP3 mRNA levels (A) and protein secretion (B) in IL-1β stimulated SW1353 cells. Cells were stimulated with IL-1β (10 ng/mL) in the presence of different concentrations of glucosamine, chondroitin and the combination of both test compounds for 24 h. Cell culture supernatants were collected and the amount of MMP3 secreted by SW1353 cells was measured by specific ELISA. Results for MMP3 mRNA are calculated in relation to β-actin and compared to IL-1β-treated cells. Data are means + SEM of three independent experiments performed in duplicate. * *p *< 0.05; # *p *< 0.01; § *p *< 0.001; compared to IL-1β.

MMP3 mRNA levels were determined after co‑incubation of IL-1β (10 ng/mL) with GS, CS and the combination of GS and CS. Under the conditions investigated, CS was more potent than GS and the combination of GS and CS was more potent than CS only, in inhibiting IL-1β-induced MMP3 gene expression (Figure 4A). 

Differences in MMP3 gene expression in response to the treatments with GS and CS were also reflected on the protein level (Figure 4B). Again a combination of 500 µmol/L GS plus 500 µmol/L CS significantly decreased MMP3 secretion, whereas the single components resulted only in a moderate but not significant inhibition of IL‑1β-induced MMP3 secretion.

The induction of MMP3 can be counteracted by a combination of GS and CS more efficiently than by a single treatment with these test components. Thus, GS and CS act synergistically in inhibiting cytokine-induced MMP3 induction.

The underlying cellular and molecular mechanisms by which GS and CS may affect MMP3 have yet not been fully elucidated. Our data suggest that GS and CS affect MMP3 already on the transcriptional level which may in turn result in decreased MMP3 secretion. It has been suggested that GS may decrease the production of glycosylinositol phospholipid (GPI)-linked proteins which are crucial for the stimulation of chondrocytes by IL-1β [7,26].

The relatively high concentration of GS which was necessary for the inhibition of MMP3 gene expression and secretion may be related to a competition between GS and glucose [present in high concentration in our cell culture medium (4.5 g/L)] for entering the chondrocytes via glucose transporters [9,27]. Furthermore, GS has been shown to inhibit nuclear factor κB (NFκB)-dependent signal transduction pathway [28] which is involved in MMP3 upregulation [29]. Thus, antagonists of NFκB including GS may be effective inhibitors of the excessive destruction that is evident during chronic inflammatory conditions including oasteoarthritis [29].

**Figure 5:**
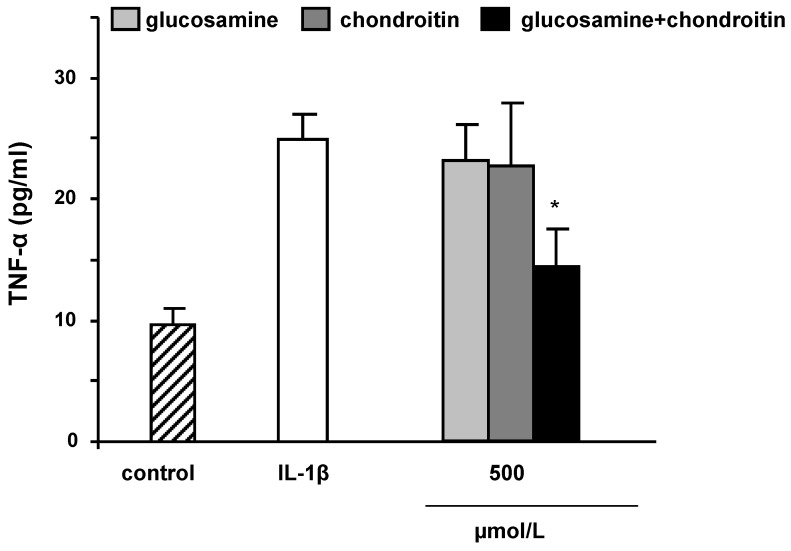
Effect of glucosamine and chondroitin on TNFα protein secretion in IL-1β stimulated SW1353 cells. Cells were stimulated with IL-1β (10 ng/mL) in the presence of 500 µmol/L of glucosamine, chondroitin and the combination of both test compounds for 24 h. Cell culture supernatants were collected and the amount of TNFα secreted by SW1353 cells was measured by specific ELISA. Data are means + SEM of three independent experiments performed in duplicate. * *p *< 0.05; compared to IL-1β.

Accordingly, it is suggested that CS may decrease NFκB nuclear translocation possibly by affecting extracellular signal-regulated kinase 1/2, p38mitogen-activated protein kinase and c-Jun N-terminal kinase activation [30]. Our results indicate that pre‑treatment of SW1353 cells with 100 µmol/L GS and 100 µmol/L CS resulted in a 20% inhibition of nuclear p65 protein levels, one subunit of NFκB (data not shown). Thus, GS and CS seem to address identical molecular targets which may explain their synergistic inhibitory activity on MMP3 gene expression and secretion. NFκB regulates TNF-α production in chondrocytes. TNF-α in turn stimulates the expression of MMP3 [31]. In the present study the inhibition of NFκB activity due to GS and CS was accompanied by a 30% decrease in TNF-α mRNA levels (data not shown) and TNF-α secretion as summarized in Figure 5. Again a combination of GS and CS was more potent than the single components *per se*.

As far as MMP3 is concerned, it should be considered that in the present study only mRNA and protein levels of MMP3 have been determined. Thus, it needs to be studied in the future whether changes in MMP3 gene expression and secretion due to GS and CS are also reflected on the activity level. Whether the inhibition of MMP3 due to the combined application of GS and CS may result in changes in extracellular matrix production needs to be also studied in the future.

## 3. Experimental

### 3.1. Cell culture

SW1353 cells, a human chondrosarcoma cell line (I.A.Z., Munich, Germany), were grown in Dulbecco’s modified eagle medium (DMEM) supplemented with 10% fetal bovine serum (FBS), 100 U/mL penicillin and 100 µg/mL streptomycin (all obtained from PAA Laboratories, Pasching, Austria). Cells were grown under standard conditions in a humidified incubator at 37 °C and 5% CO_2_. The medium was replaced every second day. For experiments, cells were seeded at an initial densitiy of 5 × 10^3^ cells/cm^2^ 24 h before the start of experiments.

### 3.2. Cytotoxicity studies

Cytotoxicity of the test substances was evaluated by the neutral red assay [32,33]. Cells were treated with increasing concentrations of IL-1β (Invitrogen, Karlsruhe, Germany) (1, 10, and 100 ng/mL), glucosamine and chondroitin (100, 500, 750, and 1000 µmol/L). After 24 h incubation the medium was replaced by medium containing neutral red (3-amino-*m*-dimethylamino-2-methylphenazine hydrochloride, DMEM, 50 µg/mL; Carl Roth, Karlsruhe, Germany) and cells were incubated for 3 h. The neutral red dye was extracted by a bleaching solution containing 50% ethanol, 1% acetic acid and 49% H_2_O. Absorbance was read at 540 nm (Labsystems iEMS Reader MF, Labsystems, Finland) and cell viability was calculated as percentage of medium treated control cells. In a second set of experiments the impact of the antioxidants ascorbic acid, α‑tocopherol, and selenium (alone and in combination) on *tert*-butyl hydroperoxide (*t*-BHP)-induced cell death was studied. SW1353 cells were incubated with increasing non‑cytotoxic concentrations of ascorbic acid (0, 10, 25, and 50 µmol/L; Carl Roth, Karlsruhe, Germany), α‑tocopherol (0, 0.1, 0.5, 2.5 µmol/L; BASF, Ludwigshafen, Germany) and selenium (0, 1, 10, 50 nmol/L; Sigma-Aldrich, Munich, Germany) (single substances and combinations) for 24 h. *t*-BHP (Acros Organics, NJ, USA) was added in different concentrations (0, 100, 200, and 500 µmol/L) for 3 h to induce cell damage/death and cell viability was determined by neutral red assay.

### 3.3. Matrix metalloproteinase-3 and tumor necrosis factor alpha gene expression and secretion

#### RNA isolation and real time PCR measurements

Cells were co-incubated with IL-1β (10 ng/mL) and 100 and 500 µmol/L glucosamine sulfate, chondroitin sulfate (Bayer AG, Leverkusen, Germany) or the combination of both (100 and 500 µmol/L of each test compound). After 6, 12, and 24 h the medium was collected and the cells were lysed by TRIsure reagent (Bioline, London, United Kingdom) according to the manufacturer’s protocol. The concentration of RNA was determined by measuring the absorbance at 260 nm. The purity was determined by the ratio of 260/280 nm using a spectrophotometer (Beckman Coulter GmbH, Munich, Germany). RNA concentration was diluted to 100 ng/µL and aliquots were stored at -80 °C until PCR analysis. Real-time quantitative PCR was performed in a Rotor Gene 6000 thermocycler (Corbett Research, Sydney, Australia) and carried out using the SensiMix™ One-Step Kit with SybrGreen detection (Quantance, Berlin, Germany) according to the manufacturer’s instructions. Primer sequences for β‑actin, MMP3 and TNFα are summarized in Table 2 below. mRNA levels of MMP3 and TNFα were related to the house-keeping gene β‑actin.

**Table 2:** Primer sequences and conditions for real time PCR experiments.

Gene	Sequence (5' - 3')	Annealing temperature
b-actin	F: GGA TGC AGA AGG AGA TCA CTG	55°C
	R: CGA TCC ACA CGC AGT ACT TG	
MMP3	F: TTT TGG CCA TCT CTT CCT TCA	59°C
	R: TGT GGA TGC CTC TTG GGT ATC	
TNF-α	F: CCC CAG GGA CCT CTC TCT A	60°C
	R: GGT TTG CTA CAA CAT GGG CTA CA	

### 3.4. MMP3 and TNFα quantification by ELISA

Secretion of MMP3 and TNFα was determined in all culture supernatants using a commercial ELISA kit (DuoSet^®^ ELISA Development System, R&D Systems, Inc. Wiesbaden, Germany) according to the manufacturer’s protocol. Optical density readings at 450 nm were performed using a microplate reader (Tecan Germany GmbH, Crailsheim, Germany).

### 3.5. Western blot analysis for p65

Following pre-treatment with test compounds for 1.5 h cells were co‑incubated with IL-1β for 3 h. Subsequently, cells were washed with ice‑cold PBS, scraped and centrifuged (800× *g*, 4 °C, 3 min). After discarding the supernatant, the remaining cell pellet was carefully resuspended in 100 µl of ice‑cold buffer A [10 mmol/L HEPES (pH 7.9), 10 mmol/L KCl, 1.5 mmol/L MgCl2, 0.5 mmol/L DTT, 0.1%, Nonidet-P40, protease inhibitor cocktail (Sigma)], and incubated on ice for 15 min. Afterwards, the homogenates were centrifuged (4,000x*g*, 4 °C, 1 min) and the supernatants were removed. The pellets were resuspended in 80 µL of ice-cold buffer B [40 mmol/L HEPES (pH 7.9), 400 mmol/L KCl, 1mmol/L DTT, 6.25% 5M NaCl, 10% glycerol and protease inhibitors], left on ice for 30 min and centrifuged (18,000x*g*, 4 °C) for 30 min. Supernatants (nuclear extracts) were removed and stored at -80°C until further analysis [34].

Protein concentrations were determined by BCA protein assay (Pierce, Rockford, USA). A quantity of protein of each sample (40 µg) was mixed with loading buffer, denatured at 95 °C for 5 min and separated on a 12% SDS-PAGE. Subsequently, the samples were transferred onto a polyvinylidene fluoride (PVDF) membrane and blocked with 5% skim milk dissolved in TBS + 0.05% Tween-20 (TBST) for a minimum of 1 h. The membrane was probed with the respective antibody (p65: 1:500; actin: 1:800) (Santa Cruz Biotechnology, Heidelberg, Germany) at 4 °C overnight. Afterwards the membrane was washed and incubated with the respective HRP-conjugated secondary antibody (anti-rabbit: 1:4,000) for 50 min. The protein bands were visualized using Pierce® ECL Western Blotting Substrate kit (Pierce) in a ChemiDoc XRS system (BioRad, Munich, Germany). Relative intensities of the bands were quantified by densitometry and expressed as ratio between target protein (p65) and loading control (actin).

### 3.5. Statistical analysis

Results are expressed as mean values with SEM. Data were tested for normal distribution and analyzed by posthoc test (Dunnett following ANOVA) or by *t*‑test. In the case of non-parametric data the Mann-Whitney *U*-test was applied. Differences were considered significant when the *p* value was ≤ 0.05. Statistical analyses calculations were performed with SPSS Version 15.0.

## 4. Conclusions

Overall our results suggest that combining natural compounds such as α-toc, AA, and Se as well as GS and CS seems to be a promising strategy to combat oxidative stress and cytokine induced matrix degradation in cultured chondrocytes. The *in vitro* data regarding the synergistic antioxidant activity of α-toc, AA, and Se as well as the synergistic inhibitory activity of GS and CS on MMP3 levels and TNF-α secretion need to be validated *in vivo*.
